# Losing Track of Time on TikTok? An Experimental Study of Short Video Users’ Time Distortion

**DOI:** 10.3390/bs15070930

**Published:** 2025-07-10

**Authors:** Yaqi Jiang, Zhihao Yan, Zeyang Yang

**Affiliations:** 1 Department of Psychology, School of Education, Soochow University, Suzhou 215123, China; 20234218055@stu.suda.edu.cn; 2 School of Educational Science, Anhui Normal University, Wuhu 241000, China

**Keywords:** time perception, distortion, short video use, duration estimation

## Abstract

Short videos’ increasing popularity and increased user engagement have sparked concerns about time perception. While studies have linked gaming or watching TV series to time loss, research on short videos’ temporal impact is scarce. This study aims to investigate the impact of short video use on time distortion (including perceptions of time for experimental tasks and weekly usage) through an experimental design. Fifty-six college students were randomly assigned to two time duration conditions (long-duration for 16 min 9 s or short-duration for 5 min 23 s). Participants in both conditions were instructed to watch short videos and read public articles for the same duration and then estimate the time duration of the tasks. Subsequently, participants completed a questionnaire about their estimated and actual weekly short video use and problematic short watching levels. The results showed that the impact of task duration on time perception was significant. Task type had no significant impact on time perception, with no notable difference in time estimation between conditions involving watching short videos and reading. The interaction between time duration and task type was not significant. Additionally, problematic short video watching and the estimated weekly short video use were not significantly related to time distortion. This study contributes to empirical research on time distortion while watching short videos, providing insights for expanding theoretical models of addictive behaviors and interventions for problematic short video use.

## 1. Introduction

Short video platforms have gradually evolved into a global cultural phenomenon, gaining popularity due to immersive experiences such as personalized content recommendations, auto-scrolling, and catchy background music (earworms) (Zhang et al., 2019; Z. Yang et al., 2021; Chen et al., 2023; Y. Yang et al., 2024), combined with durations of less than 3 min or even within 1 min, making it convenient for users to watch anytime, anywhere during fragmented periods. These unique characteristics may make users more prone to behavioral addiction (Mou et al., 2021), alter their perception of time, and even impact their physical and mental health. Watching short videos was reported to be potentially problematic or addictive, which can lead to negative consequences such as impaired ability to maintain attention (Chen et al., 2023). It was noted that “time flies when you’re having fun” (Tobin & Grondin, 2009, p. 1). Previous studies have identified that gamers can possibly lose track of time and misestimate their duration of playing (Wood et al., 2007; Tobin & Grondin, 2009; Nuyens et al., 2020). However, time perception can be dependent on the duration of the activity. Tobin and Grondin (2009) found that in video games, the tasks with shorter durations were overestimated, and the longer tasks (three times longer) were underestimated. The phenomenon whereby individuals tend to underestimate longer durations and overestimate shorter durations is referred to as the “duration effect.” This duration effect on time estimation has also been observed in other tasks, such as watching movie clips (Huang et al., 2004), origami folding (Roy & Christenfeld, 2007), essay writing (Koole & Spijker, 2000), and reading (Pezzo et al., 2006). Consequently, it is necessary to investigate whether this phenomenon also exists when individuals watch short videos. Furthermore, time perception was also found to be different in various activities such as gaming, pornography use, and binge-watching. Gamers tended to overestimate their duration of playing, and time was perceived as passing faster during exposure to TV series and pornography (Cervigón-Carrasco et al., 2023). However, few studies have explored whether there are differences in time perception between watching short videos and other activities, as well as what those differences might be. A study by Y. Yang et al. (2024) revealed that participants exhibited a tendency to overestimate the length of time spent on both short video watching and general academic tasks. Furthermore, the frequency of daily short video watching was a positive predictor of such overestimation. The conclusions of this study still require further validation, and it is noteworthy that the participants in this study only estimated a 15-min task. The different task durations might affect time perception (Tobin & Grondin, 2009). Therefore, it is necessary to further consider task duration on this basis and explore the interaction between time duration and task type to fill the research gap in the field of short video consumption. This study aims to further explore whether individuals with different daily use patterns or problematic use would estimate time duration differently when confronted with video watching and other tasks of varying lengths, as well as whether their self-perceived and actual short video watching affect such time perception. The present study aimed to examine the influence of task type and task length on individuals’ time perception. The present study investigated both specific time distortions related to short video watching tasks and the broader time distortions resulting from weekly use caused by such experiences of viewing. This contributes to identifying individuals’ patterns of time perception when engaging in short video scrolling and enables the integration of time distortion mechanisms with behavioral addiction models (such as the I-PACE model), providing a basis for users to manage their time spent, as well as facilitating the design of interventions and the mitigation of the adverse effects of social media.

## 2. Literature Review

### 2.1. Problematic Short Video Watching

Earlier studies have raised concerns about the overuse of the internet or internet addiction (Young, 1998). Addictive online behaviours are always reported to have six distinct components, including salience, mood modification, tolerance, withdrawal, conflict, and relapse (Griffiths, 2009). The cognitive–behavioral model of pathological internet use proposed the necessity of distinguishing problematic/pathological internet use with specific purposes, such as online gambling and generalized internet use (Davis, 2001). Watching online videos, as one of the most popular activities on the internet, has been widely investigated as a potential problematic or addictive behavior in recent studies, such as short video watching addiction (Zhang et al., 2019; Chen et al., 2023), on-demand TV streaming addiction (Flayelle et al., 2023), binge watching addiction (Forte et al., 2021), problematic mukbang watching (Kircaburun et al., 2021), etc. Short videos, typically lasting less than one minute, have garnered widespread attention for their addictive nature, thanks to their easy-to-use designs (e.g., auto-scrolling) and personalized recommendations based on algorithms (Zhang et al., 2019; Z. Yang et al., 2021). To avoid the risk of overpathologizing daily behaviours, in the present study, we have decided to use the term problematic short video watching (PSVW) instead of short video addiction. Based on the descriptions of short video watching addiction and problematic online video watching (Zhang et al., 2019; Chen et al., 2023; Yan et al., 2023), PSVW could be defined as behaviors associated with the excessive viewing of short videos that may lead to potential functional impairments and other negative consequences.

The Interaction of Person–Affect–Cognition–Execution (I-PACE) model suggests that addictive behaviors, such as gaming disorder, gambling addiction, and compulsive buying, can be developed from early stages to later stages (Brand et al., 2019). In later stages, individuals tend to exhibit reduced inhibitory control over their craving for the specific activities (e.g., watching short videos) evoked by specific triggers (e.g., confrontation with video watching) and experience compensation more than gratification (in early stages). Such feelings of compensation can then result in the vicious loop between addictive behaviors and negative consequences (Brand et al., 2019). Overall, addicted individuals appear to constantly crave more rewards as compensation and heavily engage in a specific activity (such as watching short videos) uncontrollably.

### 2.2. Time Perception and Time Distortion

The process of time perception is defined as the transformation of stimulus time into judgmental time (Chinchanachokchai et al., 2015; Graham, 1981). Time perception serves as the foundation for individuals’ emotions and behaviors across various activities (Meissner & Wittmann, 2011). According to Fraisse (1984), time perception encompasses two distinct subjective experiences: the perception of succession, which concerns the awareness of the sequential ordering of events, and the perception of duration. The latter involves the assessment of the temporal span between two occurrences or the temporal extent of a specific event, as elaborated by Xu and David (2018). Some researchers have argued that time perception involves duration estimation and subjective judgments of how time passes, that is, the psychological perception of how quickly or slowly time appears to pass (Sucala et al., 2011; Block, 1990; Wearden, 2005). Perceived time passage may be influenced by how limited cognitive resources are allocated. People tend to perceive time as moving slowly when they have enough attention to focus on time information (Thomas & Weaver, 1975). Building on this perspective, the present study adopts Zakay’s (2014) view that time perception consists of three aspects: duration, pace, and the order of perceived and internal events. In this study, we examine the factor of time duration in the usage of short videos.

Although time itself is objectively existent, the perception of time is subjective and influenced by various factors (Chinchanachokchai et al., 2015). Internal factors, such as emotions (Bisson et al., 2009) and cognitive abilities (Liu & Li, 2020), as well as external factors like task difficulty (Block et al., 2010), stimulus presentation order (Brown, 2008), and background music (Cassidy & Macdonald, 2010), all affect time perception. Research paradigms in the field of time perception include prospective and retrospective paradigms. The prospective paradigm refers to participants knowing in advance that the task involves estimating time intervals; thus, they are consciously judging duration (Block, 1990). Conversely, the retrospective paradigm refers to participants not being aware of the need to estimate time intervals until after the target interval has ended, representing a retrospective experience (Block, 1990).

The results of studies using the prospective and retrospective paradigms for duration estimation are inconsistent. Therefore, previous research has provided theoretical explanations in terms of attentional and memory models, respectively (Sucala et al., 2011; Cervigón-Carrasco et al., 2023; Y. Yang et al., 2024). Attentional models include the processing time model proposed by Thomas and Weaver (1975), the attentional gate model (AGM) advocated by Zakay and Block (1995), and the attentional resource allocation model introduced by Zakay (1989), proposing that both temporal and non-temporal information compete for limited attentional resources (Xu & David, 2018). When individuals allocate more attentional resources to ongoing activities, the available attentional resources for monitoring time decrease, resulting in a subjective sense of time passing quickly (Sucala et al., 2011), such as during engaging entertaining activities (Watt, 1991). Conversely, when individuals have sufficient attentional resources to focus on temporal information, they perceive time as passing slowly (Y. Yang et al., 2024), such as during waiting periods.

The memory model suggests that time intervals are inferred through remembered storage size and the number of temporal markers (i.e., contextual changes) (Ornstein, 1969). When tasks consist of multiple components (Poynter, 1983) or involve task-switching (Xu & David, 2018), it can lead to an overestimation of time duration. Additionally, the “internal clock” model posits that an individual’s timing mechanism relies on an “internal clock”. Initially, a pacemaker emits pulses at a specific frequency, which are stored in an “accumulator system.” They are then transferred to a “comparator system” for comparison with the estimated duration of past similar events stored therein, ultimately determining the subjective estimation of duration (Allman et al., 2014; Wearden et al., 2017).

### 2.3. Problematic Short Video Watching and Time Distortion

Several studies have found that short video addiction is closely associated with flow experience (Qin et al., 2022; Ye et al., 2022). Flow was defined as a state of complete absorption in and focus on an activity where individuals are fully immersed and deeply involved (Csikszentmihalyi, 1990). Individuals in such a state tend to have fewer attentional resources available for tracking time due to their high engagement in activities or tasks (Tobin & Grondin, 2009). Therefore, it is necessary to explore whether problematic short video viewers, who are highly involved in watching videos, perceive their duration of watching differently since they are too engaged or in a flow state.

Previous studies have explored the relationship between certain online behaviors and time distortion. In the context of gaming, Rau et al. (2006) compared the duration estimation of expert and novice gamers, finding that the former tended to underestimate game time, while the latter tended to overestimate it. Tobin and Grondin’s (2009) research confirmed this result and observed that for the same duration, time spent playing games is often underestimated compared to reading tasks. However, these results were not in line with the studies conducted by Rivero et al. (2013) and Wood and Griffiths (2007). Turel et al. (2018) found a significant positive correlation between the degree of social media addiction and time distortion in a study focused on Facebook. Additionally, research on other online behaviors has shown that males tended to underestimate the time that they were exposed to pornography and TV series, while females exhibited the opposite pattern (Cervigón-Carrasco et al., 2023). Time perception can vary across different activities. Gamers appear to overestimate the length of their gaming sessions, while time seemed to flow more quickly when watching TV series or engaging with pornography (Cervigón-Carrasco et al., 2023). These empirical studies suggest that time perception may be distorted in certain problematic online behaviors. Although previous studies have explored the temporal perception of various online behaviors, their findings are inconsistent. Furthermore, short video platforms exhibit significant differences in content format and user interaction compared to online platforms such as gaming and other social media. These discrepancies may lead to distinct temporal perceptions among users compared to other online activities; hence, the applicability of previous conclusions in the context of short video platforms remains to be investigated. However, there is limited research investigating the impact of short video usage on time perception. A recent study confirmed that the frequency of daily short video watching serves as a positive indicator of the extent to which one overestimates the duration of both 15-min video watching and reading tasks (Y. Yang et al., 2024). Additionally, time estimation for academic reading was found to be more accurate than for short video watching (Y. Yang et al., 2024). Only one time duration (15 min) was set in the experimental tasks, failing to consider the influence of task duration and the interaction between task duration and task type. It would be interesting to know whether time distortion is different for shorter or longer tasks. However, empirical evidence for the time distortion or perception of short video watching is limited. Therefore, this study aims to further explore the temporal perception mechanisms involved in the process of watching short videos, based on previous research. It seeks to clarify both the similarities and differences in temporal perception between short video platforms and other social media, as well as to address the limitation of previous studies that did not incorporate duration as a variable.

### 2.4. The Present Study

According to the literature reviewed above, it is necessary to further investigate time estimation or time distortion while watching short videos among individuals with different levels of PSVW and short video use frequencies. It is also crucial to test their time estimation in diverse types of tasks with different task lengths. Therefore, the present study aims to explore the following research questions:

(1) How do short video users estimate time for experimental tasks (short video watching and article reading) with long and short durations, as well as weekly short video use?
(2) What is the relationship between PSVW and time distortion in experimental tasks (short video watching and article reading) and weekly short video use?
(3) What is the relationship between weekly short video use and time distortion in experimental tasks (short video watching and article reading) and weekly short video use?

## 3. Method

### 3.1. Participants

Utilizing the G*Power 3.1 software, we conducted a statistical power analysis to estimate the required sample size, which showed that a sample of 52 participants is needed for the standard criteria (α = 0.05; β = 0.8) based on a large effect size (d = 0.4). Fifty-six participants were recruited from a public and comprehensive university in Jiangsu, China. The average age of the students was 20.27 years (SD = 2.23), ranging from 17 to 25 years. Of the participants, 31 (55.4%) were female, and 26 (44.6%) were male. To guarantee that all participants had a recent history of utilizing short video apps (e.g., DouYin and KuaiShou), an a priori criterion for exclusion was implemented. The participant recruitment advertisement clearly noted that the present study only recruited people who have used short video apps in the past few weeks.

### 3.2. Experimental Design

A 2 (time duration: 16 min 9 s, 5 min 23 s) × 2 (task type: watching short video, reading articles) mixed experimental design was employed, with time duration as a between-subjects variable and task type as a within-subjects variable. According to previous studies, a duration of 15-min intervals seems to be an adequate span of time for users to accustom themselves to short videos and achieve a state of immersion or flow (Y. Yang et al., 2024; Tobin & Grondin, 2009). In addition, in a study on gamers’ time perception (Tobin & Grondin, 2009), the short time duration condition was set to one-third of the long time duration condition. Therefore, the present study set short and long task durations at around 5 min and 15 min. In order to prevent participants from accurately guessing the time duration, the present study set the long-duration condition to 16 min 9 s and the short-duration condition to 5 min 23 s. To maintain consistency with participants’ real smartphone usage, they were instructed to use their own video platform accounts to view customized short video content during the task of watching short videos. In the reading task, participants were asked to read public articles that were deemed relatively intriguing on their phones, aiming to minimize the potential impact of participants’ interests on the experimental results. All reading materials were sourced from reputable science communication WeChat public accounts in China (e.g., Guokr [science and technology review] and DingXiang Doctor [clinical and medical knowledge]), which are known for their high credibility. Two authors jointly selected articles that were of moderate length and had high page view counts.

### 3.3. Data Collection

All participants signed an informed consent form before starting the experiment. Upon arrival at the laboratory, participants were randomly assigned to either long or short time duration conditions. Participants in the long time duration condition were asked to watch short videos for 16 min and 9 s and read public articles for the same duration. Similarly, those in the short time duration condition watched short videos and read public articles for 5 min and 23 s each. A counterbalancing scheme was used to randomize the order of watching short videos and reading public articles. Participants were randomly allocated to either the long-duration or the short-duration tasks.

Following the completion of the two activities, participants were promptly asked to provide an estimation of the range of time durations for both tasks, i.e., the minimum and maximum estimated length of each duration. They were asked to fill in the following statement: “The duration of short video watching/article reading task lasted from __min__s to __min__s”. The means of the upper and lower limits of these two values were calculated for further analysis (Grondin & Plourde, 2007). To prevent participants from guessing the study’s aim and to avoid the shift from retrospective to prospective timing, time estimation was completed after the two tasks, rather than after each one.

Then, all participants were asked to complete a questionnaire, including the scales described below, demographic variables (gender, age, and grade), their estimated weekly engagement with short videos, and their authentic time investment in such content (based on their mobile phone usage data of all short video plantforms) in the past week. Researchers instructed participants to access their phone’s built-in screen time tracking feature (e.g., “Screen Time” on iOS or “Digital Wellbeing” on Android) during the session. Participants were guided to select the category for “Short-Video Apps” (specifically including Douyin [TikTok] and Kuaishou [Kwai]). They then extracted and reported their total usage duration for these apps over the past 7 days, which was recorded by the researcher. After completion, each participant was asked a question: “Did you utilize any electronic tools to estimate the duration of time?” No participants indicated that they had used their electronic devices (e.g., phones or watches) to estimate time. They were then informed about the research aims.

### 3.4. Measures

#### 3.4.1. Estimated-to-Actual Time Ratio (TE/TA) for Experimental Tasks

The essential dependent variable that reflected time perception was the ratio of estimated time to actual time, which was calculated as the quotient of the mean of the estimated time span (TE) provided by participants for completing tasks (short video watching or article reading) and the corresponding actual time duration (TA), expressed as follows: TE = (lower limit + upper limit)/2; TE/TA = TE/actual time duration (TA). This indicator can accurately and clearly reflect the magnitude and direction of time distortion for experimental tasks. When TE/TA = 1, this indicates perfect time estimation. A ratio below 1 indicates an underestimation, while a ratio above 1 indicates an overestimation. This approach was adopted from a previous study (Y. Yang et al., 2024), which is useful in estimating the direction of time distortion.

#### 3.4.2. Estimated-to-Actual Time Ratio (TE/TA) for Weekly Short Video Use

Participants were instructed to provide an estimation of their weekly short video use and actual weekly short video use (based on their smartphone usage records). The ratio of estimated to actual time was also calculated for weekly short video use to reflect the magnitude and direction of time distortion perceived by individuals over a longer timeframe. It was calculated by dividing the self-reported weekly use of short videos by the actual weekly use, which was recorded by participants’ smartphones.

#### 3.4.3. Problematic Short Video Usage Assessment Questionnaire for Adolescents

PSVW was measured using the Problematic Short Video Usage Assessment Questionnaire for Adolescents, which was adapted from the Problematic Mobile Social Media Usage Assessment Questionnaire for Adolescents (Jiang, 2018) and comprises 20 items. All the items were rated on a five-point Likert scale ranging from not at all (1) to completely (5), yielding an overall score from 20 to 100. This scale was utilized to standardize and quantify individuals’ short video usage by assigning a numerical score. A higher total score indicated a higher level of PSVW. This scale showed good reliability in the present study (Cronbach’s α = 0.838).

### 3.5. Data Analysis

During the data preprocessing stage before data analysis, we calculated the values of the “Estimated-to-actual time ratio (TE/TA),” as described above. We conducted descriptive statistics, correlation analysis, and one-sample *t*-tests to assess the deviation of the ratio of estimated time to actual time from the benchmark value of 1. We also used repeated measures ANOVA to examine the main effects of time duration and task type, as well as their interaction. Additionally, ANOVA and chi-square tests were performed to compare the differences in time estimation among participants with different levels of PSVW and weekly use.

## 4. Results

### 4.1. Descriptives and Correlations

Descriptive statistics of the study’s variables and their correlation coefficients are presented in Table 1. The ratio of estimated time to actual time for short video use was significantly correlated with the corresponding ratio for reading (r = −0.395, *p* < 0.05). The estimated weekly use was significantly and positively correlated with actual weekly use (r = 0.596, *p* < 0.05). The actual weekly use was significantly and negatively correlated with the estimated-to-actual time ratio of weekly use (r = −0.267, *p* < 0.05).

### 4.2. Time Distortion in Experimental Tasks and Weekly Short Video Use

**Figure 1:**
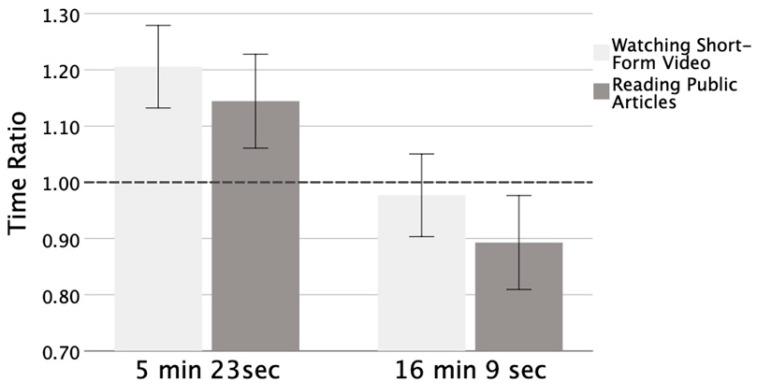
Ratio of estimated time to actual time of the watching short videos task and reading public articles task for the 5 min 23 s duration and the 16 min 9 s duration. Error bars = ±1 SE.

In order to investigate potential distortion in the perception of time during experimental tasks and weekly short video watching, we employed one-sample *t*-tests to assess the discrepancy between the ratio of estimated to actual time and 1. As shown in Figure 1 and Table 2, in the watching short videos task, for the 5 min 23 s duration, participants’ ratios of estimated time to actual time was significantly greater than 1: *t* (27) = 2.458, *p* = 0.021, 95% CI for mean difference = [0.034, 0.377]. However, for the 16 min 9 s duration, the time ratio exhibited no significant deviation from the value of 1: *t* (27) = −0.377, *p* = 0.709, 95% CI for mean difference = [−0.150, 0.103]. In the reading task, the time ratio did not exhibit significant deviation from the value of 1, regardless of the duration. Specifically, this finding was the same for both the 5 min 23 s duration (*t* (27) = 1.487, *p* = 0.149, 95% CI for mean difference = [−0.055, 0.344]) and the 16 min 9 s duration (*t* (27) = −1.587, *p* = 0.124, 95% CI for mean difference = [−0.246, 0.031]). Additionally, the ratio of the estimated time to the actual time of weekly short video use (*M* = 1.535, *SD* = 1.212) revealed a substantial overestimation, significantly surpassing the threshold of 1: *t* (95) = 3.301, *p* = 0.002, 95% CI for mean difference = [0.210, 0.859].

The results of the 2 (time durations: 16 min 9 s and 5 min 23 s) × 2 (task types: watching short videos and reading public articles) repeated measures ANOVA indicated that there was a significant main effect for time duration (*F* (1, 54) = 6.950, *p* = 0.011, *η_p_*^2^ = 0.114) but no significant main effect for task type (*F* (1, 54) = 1.287, *p* = 0.262, *η_p_*^2^ = 0.024). The time ratio of the short duration was significantly higher than the time ratio of the long duration. The interaction between time duration and task type was not significant: *F* (1, 54) = 0.031, *p* = 0.860, *η_p_*^2^ = 0.001. Based on the F-test results, we calculated *η*^2^ = 0.024 for the main effect of the task type. Using a G*Power of 3.1 (α = 0.05, two-tailed), with a correlation of r = 0.6 between time estimates across tasks, a post hoc power analysis indicated that our study achieved 74.1% power (effect size f = 0.159) to detect the observed effect.

### 4.3. Relationship Between PSVW and Time Distortion

To investigate whether PSVW is linked with time estimation for different tasks and weekly use, we divided the participants into a high-score group (*n* = 15), a moderate-score group (*n* = 26), and a low-score group (*n* = 15), using 27% at the high and low ends of the scores for the Problematic Short Video Usage Assessment Questionnaire as the cutoff points.

First, we performed separate one-sample *t*-tests to evaluate the discrepancy between the ratio of estimated to actual time and 1 for the experimental tasks, including watching short videos and reading, as well as weekly short video use for each of the three groups (high-, moderate-, and low-level problematic short video users). The findings indicated that only the ratios of estimated to actual time for weekly short video usage in the moderate score group ((*M* = 1.710, *SD* = 1.380), *t* (25) = 2.623, *p* = 0.015, 95% CI for mean difference = [0.153, 1.267]) and low score group ((*M* = 1.534, *SD* = 1.010), *t* (14) = 2.046, *p* = 0.060, 95% CI for mean difference = [−0.026, 1.093]) was significant and marginally significant different from 1. In other words, moderate- and low-level problematic short video users tended to overestimate their weekly use.

Second, we conducted a 2 (time durations: 16 min 9 s and 5 min 23 s) × 3 (PSVW groups: high, moderate, and low) two-way ANOVA. The main effect of time duration was significant for both the ratio of estimated to actual time for watching short videos (*F* (1, 50) = 5.110, *p* = 0.028, *η_p_*^2^ = 0.093) and reading (*F* (1, 50) = 4.465, *p* = 0.040, *η_p_*^2^ = 0.082). For both ratios of estimated time to actual time for engaging in the activity of watching short videos and for reading, the short-duration time ratio was significantly higher than that of the long duration. The significant main effect of group membership and the interaction between time duration and group on the ratio of estimated to actual time spent on both watching short videos and reading was not observed.

Then, we performed a 2 (task types: watching short video and reading public articles) × 3 (problematic short video use groups) repeated measure ANOVA. The results revealed that the main effect of task type (*F* (1, 53) = 0.987, *p* = 0.325, *η_p_*^2^ = 018), the main effect of group (*F* (2, 53) = 0.219, *p* = 0.084, *η_p_*^2^ = 0.008), and the interaction between them (*F* (2, 53) = 0.070, *p* = 0.932, *η_p_*^2^ = 0.003) were not significant.

To conduct nonparametric chi-square tests, we converted the continuous variable of problematic short video usage and the ratio of estimated time to actual time of watching short videos, reading, and weekly use into categorical variables. The analysis revealed no significant trends in time distortion among the three groups, either when watching short videos (*χ*^2^ (2) = 0.596, *p* = 0.742) or reading (*χ*^2^ (2) = 0.647, *p* = 0.724). The direction of time distortion for weekly use was not significant: *χ*^2^ (2) = 1.387, *p* = 0.500.

### 4.4. Relationship Between Weekly Short Video Use and Time Distortion

Likewise, to investigate whether weekly use is linked with time estimation for different tasks and weekly use, we divided the participants into a high-frequency group, a moderate-frequency group, and a low-frequency group, using 27% at the high and low ends of the scores for the estimated and actual weekly short video use as the cutoff points.

For the self-reported weekly use, first, we performed separate one-sample *t*-tests to evaluate the discrepancy between the ratio of estimated time to actual time spent and 1 for the experimental tasks, including watching short videos and reading, as well as weekly short video use for each of the three groups (high-, moderate-, and low-frequency estimated weekly short video users) (see Table 3). The findings indicated a significant deviation from 1 in the ratios of estimated to actual weekly short video usage time for both the high-frequency group ((*M* = 1.804, *SD* = 0.874), *t* (14) = 3.562, *p* = 0.003, 95% CI for mean difference = [0.320, 1.288]) and the moderate-frequency group ((*M* = 1.692, *SD* = 1.534), *t* (25) = 2.300, *p* = 0.030, 95% CI for mean difference = [0.072, 1.312]). In the moderate-frequency group (*M* = 1.206, *SD* = 0.455), the observed ratios of estimated time to actual time for the short video task (*t* (25) = 2.307, *p* = 0.030, 95% CI for mean difference = [0.022, 0.390]) were significantly different from 1. In other words, moderately estimated weekly short video users exhibited a tendency to overestimate the short video watching task and their weekly use. Self-reported high-frequency weekly users appeared to overestimate their weekly use.

Second, we conducted a 2 (time durations: 16 min 9 s and 5 min 23 s) × 3 (estimated weekly short video use groups: high, moderate, and low) two-way ANOVA. The main effect of time duration was significant for the ratio of estimated to actual time spent reading: *F* (1, 50) = 4.246, *p* = 0.045, *η_p_*^2^ = 0.078. The ratio of time spent on the short-duration task was significantly greater than that spent on the long-duration task. For watching short videos, the main effect of time duration was not significant for the ratio of estimated to actual time: *F* (1, 50) = 3.093, *p* = 0.085, *η_p_*^2^ = 0.058. The significant main effect of the group and the interaction between the time duration and group on the ratio of estimated to actual time spent on both watching short videos and reading was not observed.

Then, we performed a 2 (task types: watching short videos and reading public articles) × 3 (estimated weekly short video use groups) repeated-measures ANOVA. The results revealed that the main effects of task type (*F* (1, 53) = 0.260, *p* = 0.613, *η_p_*^2^ = 0.005) and group (*F* (2, 53) = 0.188, *p* = 0.830, *η_p_*^2^ = 0.007) were not significant. The interaction between them (*F* (2, 53) = 3.404, *p* = 0.041, *η_p_*^2^ = 0.114) was significant. The moderate-frequency group exhibited a significantly higher ratio of estimated time to actual time spent watching short videos compared to that for reading.

For the actual weekly use, the results of both the one-sample *t*-test and ANOVA were not significant.

To conduct nonparametric chi-square tests, we also transformed the continuous variables of actual weekly short video use and estimated weekly short video use into categorical variables. Using the same grouping method as described above, based on the actual and estimated (self-reported) short video weekly use, we divided the participants into three groups: high-frequency, moderate-frequency, and low-frequency. For the time ratio, the values were classified as “overestimation” when the TE/TA ratio exceeded 1, and conversely, they were labeled as “underestimation” when the TE/TA ratio was below 1.

**Figure 2:**
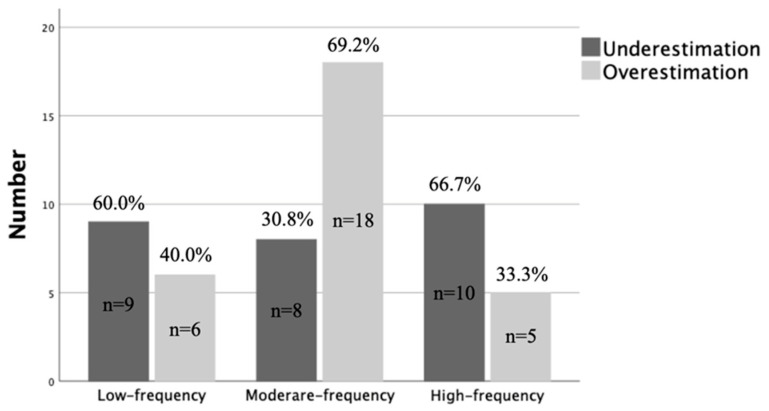
Time estimation for the short video watching task among the three self-estimated weekly use frequency groups.

The results of the chi-square tests indicated a significant discrepancy in the time distortion tendency among the three groups of estimated weekly use in the task of watching short videos: *χ*^2^ (2) = 6.049, *p* = 0.049. Figure 2 presents the distribution of participants across the two subtypes of time estimation for watching a short video, segmented into three groups. The findings revealed that approximately fifty percent of the participants overestimated and underestimated the duration of watching short videos. Those belonging to the middle-frequency group of estimated weekly use exhibited a tendency toward overestimating the length of time spent on the short video watching task. Additionally, the orientation of time distortion in the task of watching short videos among the three groups of actual weekly use was not significant: *χ*^2^ (2) = 0.678, *p* = 0.712. The orientation of time distortion in the task of reading among the three groups of both actual weekly use (*χ*^2^ (2) = 0.107, *p* = 0.948) and estimated weekly use (*χ*^2^ (2) = 0.647, *p* = 0.724) was not significant. However, the direction of time distortion of weekly use among the three groups of both actual weekly use (*χ*^2^ (2) = 7.267, *p* = 0.026) and estimated weekly use (*χ*^2^ (2) = 6.885, *p* = 0.032) was significant.

## 5. Discussion

### 5.1. Summary of the Findings

The present study found that participants tended to overestimate the duration of watching short videos at a shorter time duration of 5 min and 23 s, compared with the duration of 16 min and 9 s. However, there was no significant time distortion observed in the duration estimation of reading. Participants also tended to overestimate their weekly short video usage. The results revealed a significant main effect of time duration, indicating that the estimated-to-actual ratio for the short-duration condition was significantly higher than that for the long-duration condition, while there was no significant main effect of task type. PSVW had a minimal impact on time distortion, with participants in the moderate-score and low-score groups tending to overestimate their weekly usage. The estimation of weekly short video use did not have a significant impact on time distortion, but participants in the high- and moderate-frequency groups exhibited a tendency toward overestimating their weekly engagement with short videos. Overall, the direction of time distortion varied across different task types and groups.

### 5.2. Theoretical and Practical Implications

According to the findings of the present study and previous studies, the time distortion or perception of certain activities might be affected by multiple factors, including the time duration of the task, the task type (watching or reading), and individual differences (e.g., frequency of that daily behaviour) (Tobin & Grondin, 2009; Cervigón-Carrasco et al., 2023; Y. Yang et al., 2024). The present study found that the participants’ overall time estimation was close to 1, but with relatively large standard deviations (short video: ±0.402; reading: ±0.456), indicating significant individual differences in time perception. This variability may stem from differences in attentional allocation, cognitive load, or prior usage habits among participants, which warrants further investigation in future studies. Users tended to overestimate the length of a short session of viewing short videos lasting about 5 min, but underestimated a longer session lasting about 16 min. This is in line with previous studies about gamers, in which individuals overestimated the shorter tasks and underestimated the longer tasks (Tobin & Grondin, 2009). Such findings can be explained by the classic effect of interval segmentation in time duration judgment; this effect suggests that individuals tend to estimate the length of time based on the number of events in their memory (Poynter, 1983). In other words, from the perspective of memory models, the high variability of content in short videos increases the number of events in memory, leading the participants to overestimate the actual time elapsed. Furthermore, the Attentional Resource Allocation Model (Zakay, 1989) can also explain this phenomenon. When viewing short videos, highly engaging content stimulates and occupies a significant amount of an individual’s cognitive resources, leading them into a state of immersion. Consequently, the allocation of attention to time information decreases, causing individuals to lose track of time’s passage, ultimately resulting in an overestimation of time. However, unlike the present study, Y. Yang et al. (2024) only set one task duration of 15 min and found that participants overestimated the 15-min short video watching task. The authors explained that the frequent changes and switches of the short videos might affect the participants’ retrospective time estimation, although they were immersed in watching videos, as prospective and retrospective time perception are different processes. With a similar time duration, the present study observed an underestimation for the longer task, although it was not significant. The participants in the present study might have been in a flow state and forgotten the time they spent watching, resulting in underestimation (retrospective). Overall, it is important that studies on the time distortion and perception of daily activities consider task length carefully and be aware of the difference between prospective and retrospective time estimation.

In addition to task time duration, task type (e.g., watching videos or reading) is another key element that affects time perception. The present study found no significant main effect of task type, i.e., time distortion for video watching and reading was similar. This is in line with previous findings that time distortion could be generalized to other daily activities such as reading (Y. Yang et al., 2024). Meteier et al. (2025), in their study utilizing a short-duration task (7.5 min), similarly found no significant differences in time perception between digital and non-digital activities. This suggests that task type may not be a core factor in time distortion. This lack of difference could be attributed to digital tasks potentially consuming fewer cognitive resources than non-digital tasks, while the underlying mechanisms of time perception remain comparable across both types. However, Tobin and Grondin (2009) reported that participants perceived gaming tasks to be shorter than reading tasks. They believe that gaming requires more mental effort and cognitive resources than reading and explained such results based on an attention-based hypothesis that time estimation could be shorter when mental workload is higher (Brown & Boltz, 2002; Sucala et al., 2011). Since watching short videos and playing games are different activities, gaming requires multi-sensory coordination and active attention, whereas browsing videos involves passive reception without conscious effort. Due to the differing allocation of cognitive resources by individuals when they are engaged in these two tasks, it is not surprising that the findings are contradictory. In terms of such a difference, a recent study investigated time distortion for three different activities (video gaming, pornography use, and TV series watching) and found that gaming time was overestimated, but pornography and TV series watching were perceived as passing fast (Cervigón-Carrasco et al., 2023). They noted that the reason for such a difference could potentially be attributed to the gaming-related cues present in the experimental videos, which failed to replicate authentic gaming scenarios involving in-game interactions, thus lacking an impact on time perception (Cervigón-Carrasco et al., 2023). It is clear that the frequent, intense multisensory coordination involved in gaming and sitting in front of a screen watching videos will definitely result in different time perceptions. This indicates that when estimating the duration of different activities, the complexity and authenticity of a particular activity will probably affect time perception.

The impact of PSVW and weekly short video use frequency on time distortion is another key concern of the present study. However, in line with previous evidence (Cervigón-Carrasco et al., 2023), this study did not identify a significant main effect of PSVW on time distortion. Only the moderate and low PSVW groups overestimated their weekly short video use. However, Turel et al. (2018) identified a significant effect of social media addiction on the upward time estimation of a 20-min survey task about controlling Facebook use. Again, as discussed above, the task type and duration in such experiments are key components that could affect time perception. It is thus necessary to further check if problematic behaviors (e.g., PSVW, gaming disorder, or social media addiction) can lead to time distortion in corresponding authentic tasks (e.g., watching short videos, playing games, or scrolling on social media).

Furthermore, previous studies found that the frequency of daily short video use positively predicted the overestimation of video watching tasks (Y. Yang et al., 2024). Conversely, the present study revealed that self-reported frequent users overestimate their weekly short video use, but not the video watching tasks conducted in the lab. Only the self-reported moderate users overestimate their video watching task durations. Those who radically believe that they spend a very long or short time watching videos, compared to moderate users, might have different perceptions of time. They tended to underestimate the time spent on short video watching tasks. This also suggests that similar time perception experiments may need to consider extreme tendencies related to how individuals evaluate their own behavior frequency, including both overestimators and underestimators.

The present study and previous studies have shown that it is possible to add time distortion or biased time perception to the theoretical models of addictive behaviors, such as the I-PACE model. A review of time perception and impulsivity suggests that behavioral addiction could be a good model for understanding the link between impulsivity and time perception, and addicts are more likely to have time distortion and behave impulsively (Paasche et al., 2019). Based on experimental evidence, Turel et al. (2018) also suggest that time distortion might be a useful marker of social media addiction. It is thus necessary to further discuss whether time distortion could be one component somewhere in the loop of addictive behaviors in the I-PACE model (Brand et al., 2019).

This study also offers theoretical insights for the prevention and intervention of short video addiction. For instance, short video platforms can refine their autoplay features and personalized recommendations to diminish users’ unconscious attention. Users can set usage duration reminders to help themselves calibrate their perception of time and avoid prolonged immersion in flow states. Additionally, schools can introduce courses related to time perception and time distortion, aiding students in recognizing the reasons behind time distortion and learning how to mitigate its effects.

### 5.3. Limitations

There are limitations that need to be noted. First, the short video watching tasks were conducted on the participants’ own smartphones. Although such a design is good for imitating the real daily behavior of short video watching, it is possible for the users to notice the time on their phones. However, all participants reported that they did not use their phones to perceive or record time because they were not aware of the research aim. Such a limitation might not affect the results. Another potential limitation might be the generalizability of the sample of university students. The results might not represent the other groups, such as the elderly or adolescents. Furthermore, the groupings in the experiment could create artificial distinctions at group boundaries, which may be a limitation. However, we sought to address this issue by randomly assigning participants to different duration groups.

One of the challenges during the experiment was that participants might have unintentionally seen the time on their smartphones. To prevent participants from guessing the study’s purpose, we did not block the time display on their smartphones. As noted previously, at the end of the experiment, all participants were asked whether they had used electronic tools to estimate time. None of the participants reported using their phones or watches for this purpose. However, this challenge should be considered in future studies replicating the present experiment.

### 5.4. Future Works

Future studies could replicate similar experiments in a larger group of participants of different ages. Additionally, in light of the present study and the study by Cervigón-Carrasco et al. (2023), further investigations are needed to compare the time distortion between different video watching behaviors, including short video watching, binge watching, live streaming, and documentary or scientific talk videos, using authentic tasks. Studies could also compare the time distortion of video watching and more action-demanding activities such as gaming or gambling. For the materials used in reading tasks, future studies could conduct pre-testing and/or gather participant feedback to better control these aspects.

Furthermore, the information processing mechanisms underlying prospective and retrospective time estimation differ (Tobin et al., 2010), which may lead to varied outcomes. Therefore, future research could consider the impact of different time estimation paradigms on the perception of short video duration and compare prospective and retrospective time estimations between watching short videos and other daily life activities. The neural mechanism underlying differences in the two types of time estimation can also be explored in conjunction with brain imaging techniques, such as fMRI.

Lastly, building upon previous research, the current study compared differences in time perception when watching short videos of varying durations. Future research could expand upon this foundation by incorporating additional duration groups to explore individual differences in time perception under conditions of longer or shorter durations, as well as to compare whether this process of change is similar to that observed under other task conditions. In the experiment, participants’ emotional states were not measured, despite evidence suggesting that emotion is one of the key factors influencing time perception (Cui et al., 2023). Future studies may consider incorporating emotional scales as covariates.

## Figures and Tables

**Table 1:** Descriptive data and correlation coefficients between study variables (*n* = 56).

	*M*	*SD*	1	2	3	4	5	6
Problematic short video usage	64.946	10.788	1					
TE/TA (short video)	1.091	0.402	−0.038	1				
TE/TA (reading)	1.019	0.456	−0.005	0.395 **	1			
Estimated weekly use (in minutes)	937.16	677.202	−0.087	−0.055	0.26	1		
Actual weekly use (in minutes)	778.55	553.849	0.106	−0.102	0.091	0.596 **	1	
TE/TA (weekly use)	1.535	1.212	−0.114	0.091	0.034	0.201	−0.398 **	1

Note: *M* = mean; *SD* = standard deviation. ** *p* < 0.01. Significant correlations were in bold.

**Table 2:** One-sample *t*-tests for TE/TA across different task types and durations.

Task Type	Time Duration	TE/TA (*M* ± *SD*)	*t*	*p*	95% CI
Short video	5 min 23 s	1.206 ± 0.443	**2.458**	0.021 *	[0.034, 0.377]
Short video	16 min 9 s	0.977 ± 0.326	−0.377	0.709	[−0.150, 0.103]
Reading	5 min 23 s	1.144± 0.514	1.487	0.149	[−0.055, 0.344]
Reading	16 min 9 s	0.893 ± 0.357	−1.587	0.124	[−0.246, 0.031]
Weekly use	-	1.535 ± 1.212	**3.301**	0.002 **	[0.210, 0.859]

Note. * *p* < 0.05, ** *p* < 0.01. Significant differences were in bold.

**Table 3:** One-sample *t*-tests for TE/TA across estimated and actual weekly use groups.

Construct	Group	TE/TA (*M* ± *SD*)	*t*	*p*	95% CI
Estimated weekly use	High-frequency group	1.804 ± 0.874	**3.562**	0.003 **	[0.320, 1.288]
	Moderate-frequency group	1.692 ± 1.534	**2.300**	0.030 *	[0.072, 1.312]
	Low-frequency group	0.993 ± 0.606	−0.047	0.963	[−0.343, 0.328]
Actual weekly use	High-frequency group	0.971 ± 0.502	−0.227	0.823	[−0.308, 0.249]
	Moderate-frequency group	1.233 ± 0.692	1.720	0.098	[−0.461, 0.512]
	Low-frequency group	2.621 ± 1.701	**3.693**	0.002 **	[0.680, 2.563]

Note. * *p* < 0.05, ** *p* < 0.01. Significant differences were in bold.

## Data Availability

The data are not publicly available due to privacy concerns.
